# Metabolomic Profiling Reveals Social Hierarchy-Specific Metabolite Differences in Male *Macrobrachium rosenbergii*

**DOI:** 10.3390/ani15131917

**Published:** 2025-06-29

**Authors:** Liping Li, Dayan Hu, Jiongying Yu, Xingyu Zheng, Miaoying Cai, Quanxin Gao, Shaokui Yi

**Affiliations:** 1 School of Life Sciences, Huzhou University, Huzhou 313000, China; llp6589@foxmail.com (L.L.); yujiongying@outlook.com (J.Y.); z1791763704@163.com (X.Z.); gaoqx2008@163.com (Q.G.); 2 Huzhou Academy of Agricultural Sciences, Huzhou 313000, China; hudayan1982@163.com; 3 Jiangsu Shufeng Prawn Breeding Co., Ltd., Gaoyou 225654, China; caimiaoyingji@126.com

**Keywords:** *Macrobrachium rosenbergii*, metabolomics, social hierarchy, aquaculture

## Abstract

The giant freshwater prawn (*Macrobrachium rosenbergii*, GFP) is a highly valuable crustacean species in global aquaculture. However, a social hierarchy exists among the distinct male morphotypes, specifically blue-clawed males (BC), orange-clawed males (OC), and small males (SMs). In this study, to identify the specific metabolites among BC, OC, and SM, hemolymph samples were collected for the untargeted liquid chromatography–mass spectrometry metabolomics (LC–MS). A total of 172, 546, and 578 significantly different metabolites (SDMs) were identified in OC vs. BC, SM vs. BC, and SM vs. OC, respectively. Notably, creatine and glutamate in BC males likely enhance their aggressive behavior through improved energy metabolism. In the SM group, the up-regulation of prostaglandin E3, testosterone, and arachidonic acid may lead to premature gonadal maturation and enhance immunity. Serotonin, Glu-Pro, and pentanoylcarnitine detected in OC males reflect their physiological need for rapid growth and adaptation to social behaviors. In the SM group, the up-regulation of prostaglandin E3, arachidonic acid, and testosterone may promote premature gonadal maturation and enhance immunodominance. These findings will enhance the understanding of the physiological basis of social hierarchy formation in male GFPs from a metabolomics perspective.

## Simple Summary

*Macrobrachium rosenbergii* males showed a social hierarchy of three morphotypes: blue-clawed males (BC), orange-clawed males (OC), and small males (SMs), arranged in descending order of social hierarchy. These morphotypes exhibit significant differences in growth performance and market value, which directly affect aquaculture economic efficiency. This study explored the reasons for the phenotypic differences of male *M. rosenbergii* from the perspective of metabolomics. The results showed that glutamate, prostaglandin E3, testosterone, arachidonic acid, serotonin, pentanoylcarnitine, and other metabolites were significantly different in different castes. In the future, it is possible to adjust the proportion of different morphotypes by adjusting key metabolic pathways or optimizing the aquaculture environment (such as feed formula and stocking density), thereby improving the growth performance and economic benefits of aquaculture production.

## 1. Introduction

The giant freshwater prawn (*Macrobrachium rosenbergii*, GFP) is a highly economically valuable crustacean species in global aquaculture, primarily distributed in tropical and subtropical regions [1]. The production of *M. rosenbergii* in China in 2023 was 196,374 tons, an increase of about 10.42% compared with 2022 [2]. Native to the Indo-Pacific region, this species has emerged as a preferred alternative to marine shrimp due to its strong adaptability to freshwater environments and high nutritional value [3,4]. *M. rosenbergii* plays a crucial role in enhancing food security and supporting livelihoods, particularly in developing countries where aquaculture is a significant economic activity [5]. However, the existence of male social hierarchies, especially the behaviors of cannibalism and fighting, restricts the high-density aquaculture of *M. rosenbergii*.

**Figure 1:**
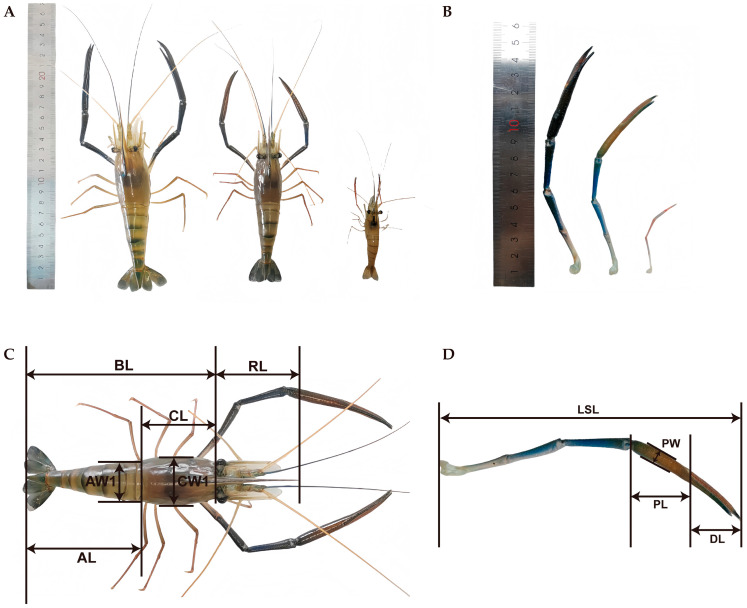
Different morphological types of male *M. rosenbergii* and phenotypic traits analyzed in this study. (**A**) Three male phenotypes of the GFP individuals. (**B**) The variation in claw color morphology. From left to right are blue claw (BC), orange claw (OC), and small male (SM). (**C**) Phenotypic traits of *M. rosenbergii* individuals. BL: body length, RL: rostrum length, CL: carapace length, AW1: abdominal width, CW1: carapace width, AL: abdominal length. (**D**) Second leg of the *M. rosenbergii* individuals. LSL: length of the second leg, PW: palm width, PL: palm length, DL: dactylus length.

Social hierarchy is a ubiquitous phenomenon in animal ethology [6,7], with the male social hierarchy of *M. rosenbergii* exhibiting typical morpho-behavioral polymorphism. This hierarchical differentiation is primarily manifested through three distinct morphotypes (Figure 1A,B): the dominant blue-clawed (BC) males (largest body size with vivid blue chelipeds), intermediate-sized orange-clawed (OC) males, and subordinate small males (SM) with translucent chelipeds [8,9]. The dominant status of BC males confers priority access to critical resources (including habitats, food, and mates), with such resource monopolization significantly enhancing their reproductive success [10,11]. Notably, maintaining dominance requires sustained energetic investments, manifested through frequent ritualized displays and direct aggressive behaviors [12,13]. Transitional males (OC) are moderately aggressive and have reproductive potential. In contrast, SM males have evolved alternative reproductive tactics [14], and they display “precocious” characteristics, possessing the smallest body size. The SM males reflect a pattern of rapid sexual maturity accompanied by limited growth. These subordinates employ sneaker mating strategies by minimizing direct competition with dominant males and selecting concealed mating opportunities, thereby improving their reproductive success. This strategic differentiation exemplifies the reproductive investment trade-off theory in crustaceans, where distinct morphotypes develop specialized adaptive strategies in mating behavior, energy allocation, and risk aversion. In addition, sexual selection pressure plays a pivotal role in maintaining this polymorphic system.

From a behavioral ecology perspective, vivid coloration, territoriality, and courtship behaviors are inextricably linked to social hierarchy, potentially serving as visual signals of social status [15,16]. Previous research indicates female preference for male morphological traits, particularly cheliped coloration [17], which may further strengthen morpho-behavioral associations through Fisherian runaway selection mechanisms. However, observed variations in morphotype-hierarchy correlations across populations suggest that environmental factors (e.g., population density, resource availability) may modulate social structure through phenotypic plasticity. Experimental evidence has confirmed that *Procambarus clarkii* individuals reared at different densities exhibit behavioral plasticity [18]. From an aquaculture perspective, understanding this polymorphism holds significant implications for optimizing aquaculture practices. Regulating morphotype proportions in cultured populations can effectively enhance both growth performance and reproductive efficiency. Furthermore, implementing differentiated management strategies (e.g., size-grading, environmental enrichment) tailored to specific morphotype requirements may reduce inter-individual aggression and mitigate growth inhibition caused by social stress. These measures hold practical value for achieving sustainable development in *M. rosenbergii* aquaculture.

The social hierarchy has been widely observed in animal behavior studies. Through metabolic regulatory mechanisms, this hierarchy has not only modulated fundamental behavioral repertoire, including aggression and courtship strategies, but may also profoundly influence energy allocation patterns, ontogenetic development, and reproductive fitness [19,20]. In recent years, the relationship between metabolism and social behavior has emerged as a significant research focus, as metabolism, a core indicator of physiological conditions, directly reflects the energetic costs underlying social interactions [21,22,23]. The metabolome, which serves as a direct reflection of an organism’s physiological state, offers unique insights into energy allocation, nutrient utilization, and the regulation of social behavior. Specifically, dominant individuals typically demonstrate enhanced metabolic efficiency due to preferential access to resources, while subordinate individuals often exhibit impaired energy metabolism as a result of chronic stress exposure [24]. Furthermore, dominant individuals exhibit significant advantages in both lipid metabolism and amino acid biosynthesis, potentially enhancing their competitive ability and reproductive success [25]. Interestingly, metabolomic analyses of the Chinese mitten crab (*Eriocheir sinensis*) demonstrate that N-acetylglucosamine-6-phosphate released during molting functions as a cannibalism signal, with this chemical communication mechanism directly modulating intraspecific social dynamics [26]. In addition, metabolic reprogramming may represent a host adaptive response to social stressors, particularly status competition. Consistently, studies in the highly social cichlid fish *Astatotilapia burtoni* demonstrate that dominant males exposed to social stress exhibit reduced oxidative DNA damage (8-OHdG) levels [27]. In crustaceans, the relationship between social hierarchy and metabolism manifests at multiple levels. At the physiological level, dominant individuals establish status through aggression, a process requiring rapid energy mobilization via glycogenolysis and lipid metabolism. For instance, fighting behavior in *Portunus trituberculatus* correlates with metabolic variation, where bold individuals exhibit higher metabolic rates during competition [28]. From a nutritional perspective, hierarchical differences influence allocation strategies. The dominant rainbow trout develop greater anabolic activity (e.g., lipid accumulation), whereas subordinates display elevated catabolism, suggesting status-dependent metabolic reprogramming [29]. Furthermore, environmental stressors (e.g., hypoxia and heavy metal pollution) may exacerbate metabolic imbalance through mitochondrial dysfunction or ferroptosis induction [30], thereby amplifying the physiological impacts of social hierarchy. The metabolic division of labor observed in arthropods offers a unique perspective for investigating social hierarchy. In eusocial insects like ants and bees, distinct metabolic differentiation exists between lean foragers and lipid-rich nest workers [31,32], a phenomenon potentially regulated by conserved nutritional signaling pathways (e.g., insulin/TOR pathway) [33,34]. Although crustaceans lack eusociality, population-level metabolic heterogeneity (e.g., energy reserve fluctuations during molting cycles) may correlate with hierarchy-related behavioral differences.

Crustaceans exhibit distinct metabolic characteristics during their growth and development, which are intricately linked to their physiological states, behavioral patterns, and environmental adaptability. However, the key metabolites and regulatory mechanisms driving the differentiation of male morphotypes in *M. rosenbergii* remain poorly elucidated. This study aims to systematically investigate the metabolite profiles and critical metabolic pathways associated with the three male morphotypes of *M. rosenbergii*. The results of this study would offer novel insights into the interplay between social hierarchy and metabolic patterns in crustaceans, providing a foundation for future research into the molecular mechanisms governing these processes.

## 2. Materials and Methods

### 2.1. Ethics Statement

The experimental protocols concerning animal care and tissue collection received approval from Huzhou University, adhering to the guidelines for laboratory animals set forth by the Ministry of Science and Technology in Beijing, China. Furthermore, all measures were implemented to mitigate animal suffering.

### 2.2. Prawn Cultivation and Sample Collection

The *M. rosenbergii* individuals utilized in this study were sourced from a single-family cohort bred in one batch at the GFP Breeding Center. A total of 10,500 juveniles with uniform size (body length of 3.43 ± 0.78 cm) and developmental stage (43-day-old) were selected for the culture experiment. The culture experiment was conducted in 12 net cages, each with an area of 25 m^2^, deployed in a single outdoor pond. Individuals from the same cohort of the *M. rosenbergii* family were randomly allocated into four experimental groups, with stocking densities established at 20, 30, 40, and 50 individuals per square meter, respectively. Three biological replicates were established for each stocking density treatment, which was designed as 20-1–20-3, 30-1–30-3, 40-1–40-3, and 50-1–50-3, respectively (Table S1). Water quality parameters were determined using an MR2003 electronic colorimeter (Nanjing Tean Electronics Co., Ltd., Nanjing, China) with the standard colorimetric method. Throughout the culture period, water quality parameters were rigorously maintained, including dissolved oxygen levels exceeding 5 mg/L, a pH range of 7.0–9.0, and ammonia nitrogen concentrations below 0.3 mg/L. The prawns were fed a commercial diet (Jiangsu Fuyuda Grain Products Co., Ltd., Gaoyou, China; crude protein content ≥ 38%, crude fat content ≥ 5%, crude fiber content ≤ 7%) 2–3 times daily, with the feeding regimen being adjusted according to environmental conditions (weather and water temperature) and growth performance. The feeding protocol was reevaluated and modified every 15 days throughout the 100-day culture period. Upon completion of the experimental trial, key performance indicators, including production yield and survival rate, were determined. Additionally, 18 phenotypic traits were measured (Figure 1C,D), including body weight (BW), body length (BL), carapace length (CL), carapace width (CW1), carapace depth (CD), abdominal length (AL), abdominal width (AW1), abdominal depth (AD), carapace weight (CW2), weight of the second leg (WSL), abdominal weight (AW2), side panel depth (SPD), side panel width (SPW), palm length (PL), palm width (PW), dactylus length (DL), rostrum length (RL), and length of the second leg (LSL).

### 2.3. Sample Collection

After a 100-day culture period, *M. rosenbergii* reached sexual maturity. According to the data analysis conducted after the harvest, groups 30-1 and 30-3, which were subjected to the same stocking density, exhibited similarities in survival rates and the proportions of male morphological types (Table S1). We collected a total of 24 hemolymph samples from two treatment groups (30-1 and 30-3), with 8 technical replicates per male morphotype (BC, OC, and SM). For hemolymph collection, *M. rosenbergii* individuals were selected and anesthetized on ice to minimize stress and movement. Hemolymph was aseptically withdrawn from the pericardial sinus using a sterile 1 mL syringe fitted with a 25-gauge needle. The collected hemolymph was promptly transferred into pre-chilled 1.5 mL microcentrifuge tubes to inhibit premature coagulation. The samples were incubated at 4 °C for 1–2 h to facilitate clot formation, followed by centrifugation at 3000 rpm for 12 min at 4 °C to isolate the serum. The supernatant was carefully aspirated, aliquoted into sterile microcentrifuge tubes, and stored at −80 °C until subsequent liquid chromatography–mass spectrometry (LC–MS) analysis. All procedures were performed under sterile conditions to ensure sample integrity.

### 2.4. LC–MS Analysis

Non-targeted metabolomics analysis was performed using liquid chromatography–mass spectrometry (LC–MS; Shanghai Majorbio Bio-Pharm Technology Co., Ltd., Shanghai, China). The experiments were carried out on an ultra-high-performance liquid chromatography system coupled with a Fourier transform mass spectrometer (UHPLC-Exploris 480, Thermo Fisher Scientific, MA, USA). The system was equipped with an ACQUITY UPLC HSS T3 analytical column (100 mm × 2.1 mm internal diameter, 1.8 µm particle size; Waters, Milford, MA, USA). The mobile phase comprised solvent A (95% water, 5% acetonitrile containing 0.1% formic acid) and solvent B (47.5% acetonitrile, 47.5% isopropanol, and 5% water containing 0.1% formic acid). The injection volume was set to 3 µL, and the column temperature was maintained at 40 °C. Sample ionization was achieved using electrospray ionization (ESI) in both positive and negative ion modes. The mass spectrometry parameters were optimized as follows: a scan range of 70–1050 *m*/*z*, sheath gas flow rate of 50 Arb, auxiliary gas flow rate of 15 Arb, capillary temperature of 350 °C, spray voltage of 3.4 kV (positive ion mode) or −2.8 kV (negative ion mode), and stepped collision energies of 20, 40, and 60 eV. The full-scan mass spectrometry resolution was set to 60,000, while the MS/MS resolution was maintained at 7500.

### 2.5. Qualitative and Quantitative Analysis of Metabolites

The raw data was processed using Progenesis QI v3.0 (Waters Corporation, Milford, MA, USA) for baseline filtering, peak identification, and peak alignment. Subsequently, the software enabled the identification of feature peaks by correlating MS and MS/MS spectral data with established metabolite databases, with a mass error threshold for MS set at less than 10 ppm. The identification of metabolites was based on matching scores derived from the secondary mass spectra.

### 2.6. Differentially Expressed Metabolites Selection

In this study, the R package “ropls” (Version 1.6.2) was used for differential metabolite analysis. Initially, unsupervised principal component analysis (PCA) was performed to assess overall metabolic differences and evaluate the variability within groups. Subsequently, supervised orthogonal partial least squares-discriminant analysis (OPLS-DA) was employed to distinguish between samples of BC, OC, and SM. The OPLS-DA model effectively eliminates variability unrelated to class separation, thereby focusing more on metabolite features that contribute to intergroup differences. The quality and reliability of the model were evaluated using R^2^X, R^2^Y, and Q^2^. Here, R^2^X represents the explained variance of the independent variables (goodness of fit), R^2^Y indicates the explained variance of the dependent variables (predictive ability), and Q^2^ estimates the predictive capability through cross-validation. Values of R^2^X(cum), R^2^Y(cum), and Q^2^ closer to 1 indicate a more stable and reliable model. To avoid overfitting, a permutation test (*n* = 200) was conducted for the OPLS-DA model, and the *p*-value from the permutation test was calculated to validate the model’s significance. Based on the variable importance in projection (VIP) analysis of the OPLS-DA model, metabolites significantly contributing to the classification of different male *M. rosenbergii* samples were identified. Further screening using a *t*-test (*p*-value < 0.05) and VIP values (VIP > 1) was performed to determine the significantly differential metabolites (SDMs). In each comparative analysis, up-regulation and down-regulation were defined in relation to the former group compared to the latter.

## 3. Results

### 3.1. Significant Morphological Advantages in BC Males

**Figure 2:**
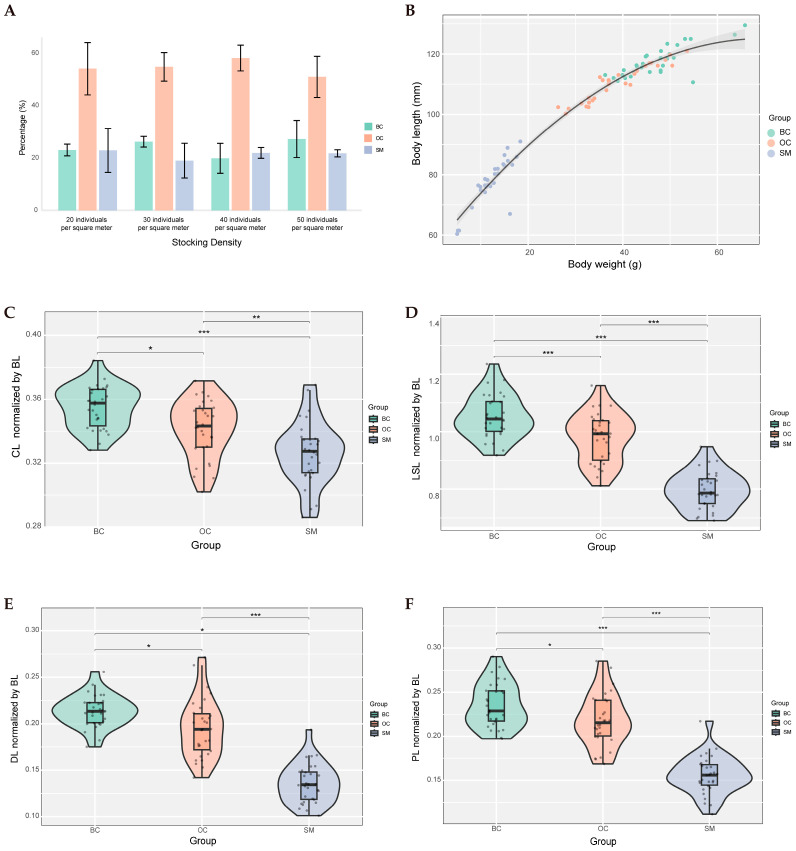
The morphological differences among male morphotypes in *M. rosenbergii*. (**A**) Morphological proportions of male *M. rosenbergii* under different stocking densities. (**B**) Binomial regression curves for body weight and body length by different morphotypes. Solid line: fitting curve, shadow area: 95% confidence interval. (**C**) Morphological differences in CL among three male castes. (**D**) Morphological differences in LSL among three male castes. (**E**) Morphological differences in DL among three male castes. (**F**) Morphological differences in PL among three male castes. BL: body length; CL: carapace length; LSL: length of the second leg; DL: dactylus Length; PL: palm Length. Statistical significance was denoted as follows: *** (*p* < 0.001), ** (*p* < 0.01), and * (*p* < 0.05).

After 100 days of culture, *M. rosenbergii* reached sexual maturity and were harvested for phenotypic trait measurements. The survival rates of *M. rosenbergii* under different stocking density treatments were 56.93% ± 12.89%, 49.60% ± 5.31%, 43.60% ± 3.47%, and 42.96% ± 3.49%, respectively. Additionally, the proportion of OC individuals was the highest in each group (Figure 2A, Table S1). Figure 2B reveals the differences in the various morphological types of weight–length relationship, with the SM group having the largest absolute quadratic term coefficient (−7.82) (Table S2), which may indicate that metabolic constraints drive energy reallocation to gonadal development. After data normalization, analysis of morphological traits revealed statistically significant differences among the three male morphotypes in CL, LSL, DL, and PL (Figure 2C–F). Notably, the BC morphotype exhibits longer CL, LSL, DL, and PL traits, which may confer morphological advantages that enhance social dominance in aggressive behaviors and feeding competitiveness in BC males.

### 3.2. Metabolic Profiles of the Three Male Morphotypes

Upon superimposing the base peak ion chromatograms of all quality control samples, it was observed that the chromatograms exhibited a high degree of overlap and uniform distribution in both positive and negative ion modes (Figure S1A,B). A dataset is considered to have acceptable quality when the Relative Standard Deviation (RSD) is below 0.3, and the cumulative percentage contribution of the peak exceeds 70% of the total. Furthermore, a Partial Least Squares Discriminant Analysis (PLS-DA) was conducted on the quality control samples in conjunction with all other samples, facilitating an examination of the overall distribution of each sample set and the stability of the entire analytical procedure (Figure S1C). The findings of the present study indicated that in the positive ion mode, a total of 6077 peaks were detected in the hemolymph samples of the three groups, 1103 of which could be successfully matched to corresponding compound information in the Metlin database. In the negative ion mode, a total of 6223 peaks were detected, 762 of which could be identified using the Metlin databases with corresponding compound information (Table 1).

### 3.3. Candidate Significant Differential Metabolites (Sdms) for Caste Differentiation of Male M. rosenbergii

**Figure 3:**
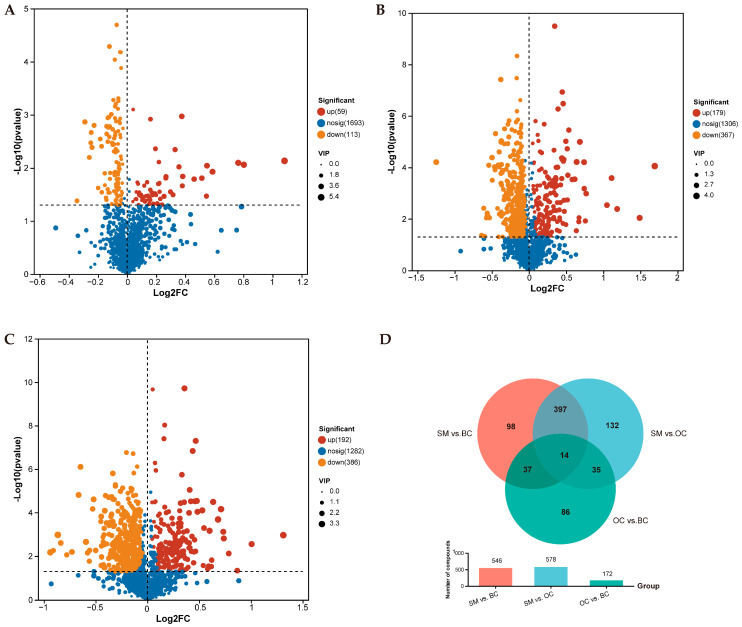
Volcano and Venn diagrams of male *M.rosenbergii* under different morphological comparison groups. Volcano plots of metabolite differences for the comparisons: (**A**) OC vs. BC, (**B**) SM vs. BC, and (**C**) SM vs. OC. Red and orange dots indicated significantly up-regulated and down-regulated metabolites, respectively, and blue dots indicated no significant difference. (**D**) The Venn diagram of metabolites of *M. rosenbergii* under different castes.

To identify specific metabolites distinguishing different male morphotypes of *M. rosenbergii* (OC vs. BC, SM vs. BC, and SM vs. OC), Orthogonal Partial Least Squares Discriminant Analysis (OPLS-DA) was used to screen for significantly differential metabolites (SDMs) with significant variations. Key metabolites were selected based on variable importance in projection (VIP) values (VIP > 1) combined with adjusted *p*-value (*p*-value < 0.05) derived from metabolite concentrations. A total of 172 SDMs were identified between OC and BC groups, including 59 up-regulated and 113 down-regulated metabolites (Figure 3A). In SM vs. BC, a total of 546 SDMs were identified, including 179 up-regulated and 367 down-regulated metabolites (Figure 3B). In addition, a total of 578 SDMs were identified in SM vs. OC, including 192 up-regulated and 386 down-regulated metabolites (Figure 3C). Table 2 lists the number of SDMs in positive and negative ion modes, respectively. The Venn diagram of the metabolites of different classes of male *M. rosenbergii* is shown in Figure 3D. In addition, a total of 14 common SDMs were evaluated for BC, OC, and SM (Table 3). The study classified the identified SDMs based on aggressive behavior, rapid growth, reproductive development, and immune response in crustaceans (Table 4). The candidate metabolites associated with morphotype differentiation in male *M. rosenbergii* include creatine, glutamic acid, prostaglandin E3, arachidonic acid, testosterone, glutaconylcarnitine, serotonin, and Glu-Pro (Table 5).

### 3.4. The Hierarchical Cluster Analysis of SDMs

**Figure 4:**
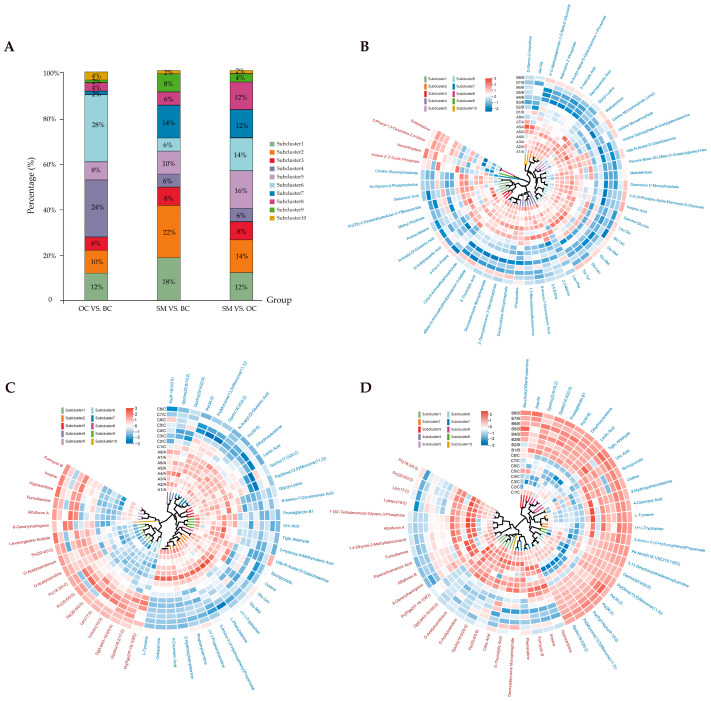
Hierarchical cluster analysis of male *M. rosenbergii* under different castes. (**A**) The proportion of the number of metabolites under sub-clusters. (**B**) The hierarchical cluster analysis in OC vs. BC. (**C**) The hierarchical cluster analysis in SM vs. BC. (**D**) The hierarchical cluster analysis in SM vs. OC. The circular dendrogram illustrates the clustering patterns of metabolites, with distinct colors representing different clusters. The outer ring displays the metabolites, where red denotes up-regulated metabolites and blue indicates down-regulated metabolites.

The top 50 SDMs in the abundance of each comparison group were selected for hierarchical clustering to evaluate the trend of SDMs in different groups (Figure 4A). The 50 SDMs between OC and BC were divided into 10 subclusters, including 6, 5, 3, 12, 4, 14, 1, 2, 1, and 2 SDMs, respectively (Figure 4B, Table S3). As shown in Figure 4C (Table S4), 10 subclusters were identified in SM vs. BC, including 9, 11, 4, 3, 5, 3, 7, 3, 4, and 1 SDMs, respectively. Concurrently, in the 10 subclusters of the SM and OC groups, they were also divided into two major clusters of metabolites, including increased or decreased metabolites in SM relative to OC (Figure 4D, Table S5).

### 3.5. The Variable Importance (VIP) Analysis of SDMs

**Figure 5:**
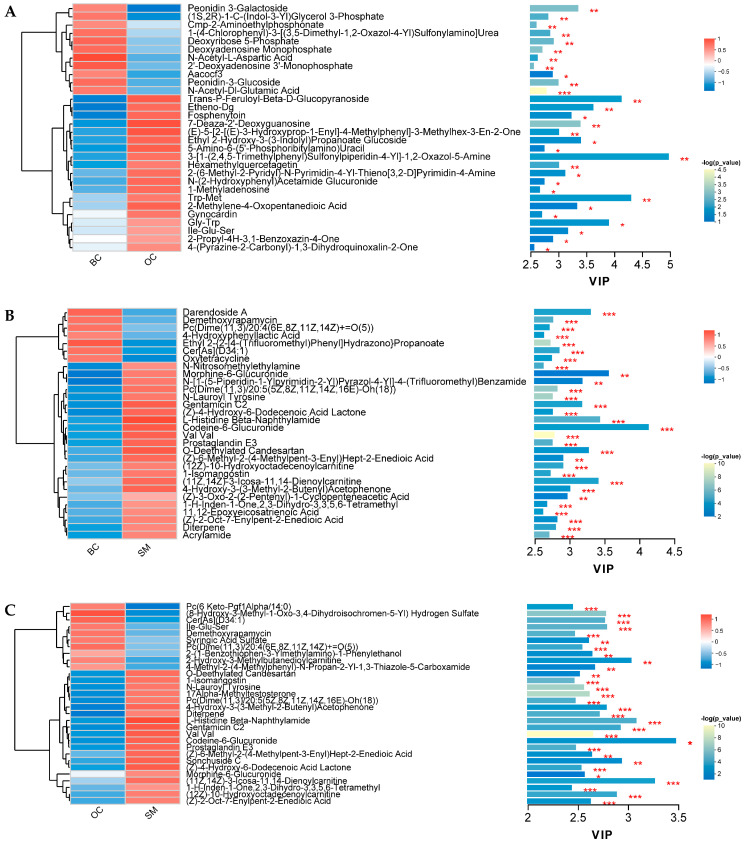
Variable importance (VIP) analysis of the top 30 SDMSs under different male phenotypes of *M. rosenbergii*. (**A**) The VIP analysis between OC and BC. (**B**) The VIP analysis between SM and BC. (**C**) The VIP analysis between SM and OC. Note: * represents *p* < 0.05, ** represents *p* < 0.01, and *** represents *p* < 0.001.

Using OPLS-DA as the model, VIP analysis was conducted on the top 30 SDMs with VIP values ≥ 1 for the three groups: OC vs. BC, BC vs. SM, and OC vs. SM. The results show that the top 10 metabolites with significant differences between the OC and BC groups are 3-[1-(2,4,5-trimethylphenyl)sulfonylpiperidin-4-yl]-1,2-oxazol-5-amine, Trp-Met, trans-*p*-feruloyl-β-D-glucopyranoside, Gly-Trp, etheno-dG, ethyl 2-hydroxy-3-(3-indolyl)propanoate glucoside, 7-deaza-2’-deoxyguanosine, peonidin 3-galactoside, 2-methylene-4-oxopentanedioic acid, fosphenytoin (Figure 5A). Figure 5B displays the variable importance in projection (VIP) bar plot of the top 30 SDMs ranked by VIP scores in the comparison between SM and BC. Among the most prominently altered metabolites between SM and BC, the top 10 showing the greatest significant differences were Val Val, ethyl 2-{2-[4-(trifluoromethyl)phenyl]hydrazono}, propanoate, N-lauroyl tyrosine, PC(dime(11,3)/20:5(5Z,8Z,11Z,14Z,16E)-OH(18)), acrylamide-demethoxyrapamycin, prostaglandin E3, L-histidine beta-naphthylamide, N-nitrosomethylethylamine, cer[as](d34:1). Furthermore, VIP analysis between the SM and OC groups is illustrated in Figure 5C. The top 10 most significantly metabolites as (11Z,14Z)-3-icosa-11,14-dienoylcarnitine, PC(6 keto-pgf1alpha/14:0), 1-h-inden-1-one,2,3-dihydro-3,3,5,6-tetramethyl, 4-hydroxy-3-(3-methyl-2-butenyl), cetophenone, (Z)-2-oct-7-enylpent-2-enedioic acid, 2-hydroxy-3-methylbutanedioylcarnitine, Codeine-6-glucuronide, (Z)-6-methyl-2-(4-methylpent-3-enyl)hept-2-enedioic acid, 2-(1-benzothiophen-3-ylmethylamino)-1-phenylethanol, and syringic acid sulfate.

### 3.6. Metabolic Pathway Enrichment Analysis Reveals Morphotype-Specific Differences

**Figure 6:**
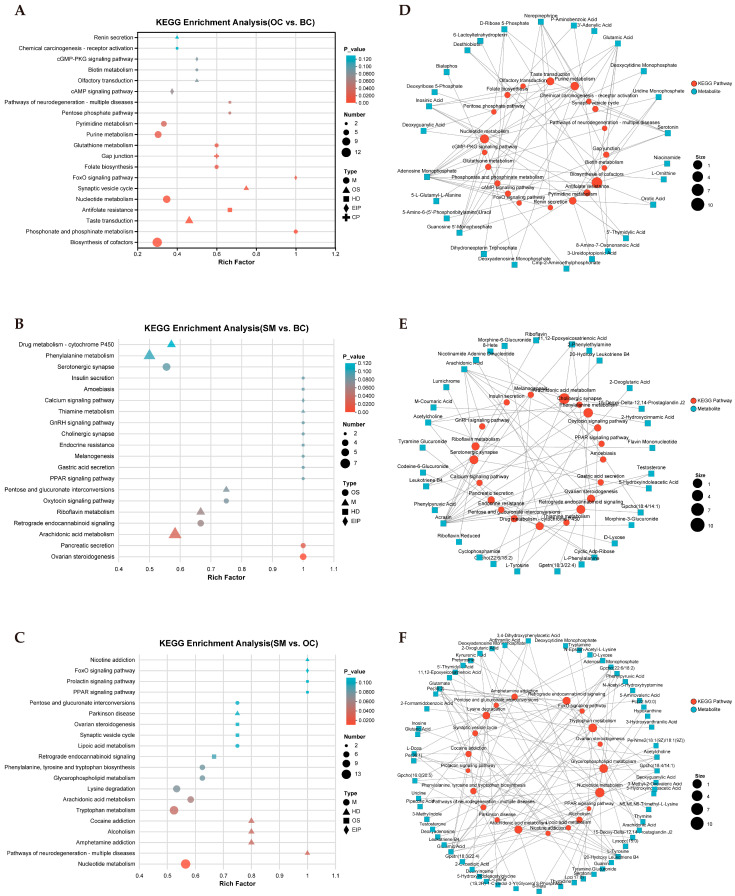
KEGG pathway enrichment analysis and metabolic network construction of SDMSs. (**A**) KEGG enrichment analysis of SDMSs in OC vs. BC. (**B**) KEGG enrichment analysis of SDMSs in SM vs. BC. (**C**) KEGG enrichment analysis of SDMSs in SM vs. OC. (**D**) Visualization of metabolite network of SDMSs in OC vs. BC. (**E**) Visualization of metabolite network of SDMSs in SM vs. BC. (**F**) Visualization of metabolite network of SDMSs in SM vs. OC. The blue square node represents the metabolite; The red circular node represents the KEGG pathway, and the size represents the number of metabolites in the pathway. The more metabolites, the larger the circular node.

To validate the metabolic pathways associated with male *M. rosenbergii* morphotype differentiation, KEGG enrichment analysis was performed for three comparative groups: OC vs. BC, SM vs. BC, and SM vs. OC. The results demonstrated that the SDMs in both the OC and BC groups were mainly enriched in metabolic pathways, such as amino acid metabolism, nucleotide metabolism, and carbohydrate metabolism (Figure 6A). A total of 105 out of the 546 SDMs identified between the SM and BC groups were enriched in specific pathways, with the top 20 pathways illustrated in Figure 6B. The results showed that the most significantly enriched pathways in SM vs. BC included pancreatic secretion and arachidonic acid metabolism. In the SM vs. OC, the most significant metabolic pathways identified were nucleotide metabolism and tryptophan metabolism (Figure 6C). Furthermore, metabolic networks were constructed for three comparison groups through Cytoscape (3.10.2) to visualize pathway details and analyze interactions among the SDMs (Figure 6D–F, Table S6). The results indicate that SDMs such as glutamate, testosterone, and arachidonic acid are enriched in multiple pathways.

## 4. Discussion

Many crustacean species exhibit social hierarchies, and male *M. rosenbergii* typically develop complex social structures within groups under shared growth conditions. These hierarchies often influence spatial resource allocation, food distribution, and breeding opportunities [45]. In aquaculture, the proportion of different morphotypes within a breeding population is closely linked to growth performance and reproductive efficiency. Metabolomics is a quantitative method used to analyze all metabolites present in an organism. Using LC–MS metabolomics technology, we identified differential metabolites (SDMs) and key metabolic pathways across distinct morphotypes of male giant river prawns.

Interestingly, a total of 14 common SDMs were evaluated for BC, OC, and SM (Table 3). 17α-Methyltestosterone (MT) is an androgen [43], and differences in its hydroxylated metabolites among different morphotypes may suggest an endocrine mechanism regulating morphological differentiation. Studies have shown that MT exhibits an excellent capability in inducing spermatogenesis [46]. SM males are the smallest in the population, yet they possess the strongest reproductive capacity. Typically, SM males employ a strategy of sneak copulation during mating between BC males and females to ensure successful reproduction [14]. Glutaconylcarnitine and hexanoyl-L-carnitine both belong to the class of acylcarnitine compounds. Acylcarnitines serve as essential carriers that facilitate the transport of fatty acids into the mitochondria for β-oxidation, and hexanoyl-L-carnitine plays a direct role in the energy generation process within the mitochondria [47]. This observation suggests that there may be significant differences in the β-oxidation of fatty acids and energy metabolism among different male morphological types. In addition, trigonelline, a biogenic alkaloid and chemical signaling molecule [48], may influence the formation of social hierarchies in crustaceans. Research has shown that trigonelline present in the urine of blue crabs (*Callinectes sapidus*) is associated with the refuge behavior of a co-occurring species, *Panopeus herbstii* [49]. This suggests that trigonelline may affect niche competition through interspecies chemical signaling.

The results of this study indicate that 172 SDMs were identified between the OC and BC groups, including creatine and glutamic acid. Creatine, a crucial molecule in animal energy metabolism [50], and glutamate, which plays a significant role in muscle energy metabolism and contraction [51], may enhance the aggressive behavior of BC males, enabling them to achieve a dominant rank. Previous studies have demonstrated that creatine levels in aquatic animals are closely linked to muscle energy metabolism [39,52], providing immediate energy support for high-intensity fighting behavior. Creatine, widely utilized as a dietary supplement, has been shown to enhance muscle protein synthesis [53]. Glutamate is one of the most abundant amino acids in the blood and can be oxidized as an energy substrate to support energy demands during exercise [54]. Additionally, glutamic acid is implicated in aggressive behavior [55]. Research has shown that acute sublethal ammonia exposure induces glutamate enzymes in the brain, thereby altering the social behavior of adult zebrafish [56]. Collectively, these findings suggest that the metabolic differences in creatine and glutamate may serve as a key physiological basis for the competitive advantage of BC males in aggressive behavior.

In the SM vs. BC groups, 179 up-regulated and 367 down-regulated differential metabolites (SDMs) were identified. Notably, the significantly elevated metabolites in SM included prostaglandin E3 (PGE3), arachidonic acid (AA), and testosterone. These findings suggest that these metabolites play crucial roles in the morphological differentiation processes of SM males. As a class of physiologically active unsaturated fatty acids, prostaglandins exert significant pheromonal functions that influence neuroendocrine regulatory mechanisms of reproductive behaviors across species [57]. Studies demonstrate that PGE can activate mating behaviors in ovoviviparous teleosts, such as black rockfish (*Sebastes schlegelii*), inducing alterations in sex steroid hormone levels [58]. Furthermore, PGE mediates neural and endocrine responses that regulate the initiation of courtship behavior in teleosts, including guppies (*Poecilia reticulata*) [41]. Arachidonic acid, an ω-6 polyunsaturated fatty acid, modulates inflammation and immune responses [59]. In zebrafish (*Danio rerio*), AA-derived 12-hydroperoxyeicosatetraenoic acid (12-HPETE) participates in inflammatory mediator-regulated TRP channels, facilitating tissue damage and repair processes [60]. Dietary enrichment with AA enhances its capacity to generate eicosanoids, demonstrating potential pro-inflammatory and oxidative consequences [61]. Testosterone, a sex steroid hormone, stimulates testicular development and spermatogenesis [62,63]. These metabolic disparities in PGE3, AA, and testosterone may confer advantages to SM males in terms of gonadal development and immune adaptation. In addition, morphological difference analysis showed that SM individuals had growth inhibition. Collectively, this may indicate that metabolic restriction drives energy redistribution to gonadal development.

Serotonin (5-HT), Glu-Pro, and glutaconylcarnitine were identified in both the SM and OC groups, with elevated levels observed in the OC group. Compared to the SM group, the increased levels of 5-HT in the OC group often result in OC individuals exhibiting superior agonistic behavior, providing evidence that the social hierarchy of OC is higher than that of SM. This finding is consistent with previous research [36,64]. Glu-Pro, along with other umami compounds (e.g., Glu-Glu and Asp-Phe), contributes to the umami characteristics of crustaceans. These compounds possess extremely low taste thresholds, significantly enhancing umami, saltiness, and lingering flavor [65,66]. Glutaconylcarnitine plays a role in the mitochondrial fatty acid β-oxidation process, and variations in its expression levels are significantly associated with fat distribution [67]. Therefore, we speculate that the notable differences in 5-HT, Glu-Pro, and glutarylcarnitine levels in OC males reflect their physiological requirements for rapid growth and social behavior.

This study indicated that SDMs are significantly enriched in pathways such as nucleotide metabolism and arachidonic acid metabolism. Nucleotides play a crucial role in metabolic processes, and the findings of this study reveal that many nucleotide metabolites exhibit significant differences among various male morphs, which may be linked to their distinct social behaviors and differences in energy metabolism. For example, in the OC vs. BC groups, the expression of adenosine monophosphate is down-regulated. Previous research has also demonstrated that larger body size, maintained through territorial behavior, is associated with a higher standard metabolic rate (SMR), with energy demands allocated for aggressive and display behaviors [68,69]. The arachidonic acid metabolic pathway (*p*-value = 0.03; leukotriene B4, arachidonic acid) is a crucial lipid signaling pathway in organisms. It generates eicosanoids with diverse biological activities through various enzymatic reactions and plays a significant role in inflammation and immune responses. These findings suggest that, despite being in a subordinate position, SM males possess certain advantages regarding immunity. These results reveal the potential mechanisms underlying male morph differentiation in the *M. rosenbergii* from a metabolomics perspective. In the future, it may be feasible to effectively regulate the proportions of different morphs by manipulating key metabolic pathways or optimizing the farming environment—such as feed formulation and stocking density—thereby enhancing growth performance and economic benefits for the farming population.

## 5. Conclusions

In this study, a 100-day cultivation of single-family GFP juveniles was carried out, and we observed that the proportion of OC individuals was the highest after sexual maturity. We established a model for the various morphotypes of male *M. rosenbergii* based on morphological indicators. The results indicated that BC males possess significantly larger body size characteristics, including longer CL, LSL, DL, and PL. These traits confer a morphological advantage, enabling them to dominate in intraspecific aggressive behavior and resource competition. A non-targeted metabolomics analysis utilizing LC–MS revealed significant differences in metabolites among the different morphotypes. A total of 6077 peaks and 6223 peaks were extracted via LC–MS in positive and negative ion mode, respectively. Notably, differential metabolites such as creatine and glutamate in BC males may enhance their aggressive behavior by improving energy metabolism. In contrast, the up-regulation of prostaglandin E3, arachidonic acid, and testosterone in SM males may play a role in regulating Premature gonadal maturation and immunity. Additionally, the differential expression of 5-HT, Glu-Pro, and pentanoylcarnitine in OC males reflects their physiological requirements for rapid growth and adaptation to social behavior. Therefore, these findings enhance our understanding of the phenotypic differentiation of male *M. rosenbergii* from a metabolomic perspective.

## Figures and Tables

**Table 1:** Number of metabolites and number of metabolites with identification information detected in both the positive and negative ion modes.

Ion Mode	All Peaks ^3^	Identified Metabolites	Metabolites in Library	Metabolites in KEGG
Positive ion mode (POS) ^1^	6077	1103	752	345
Negative ion mode (NEG) ^2^	6223	762	595	286

^1^ Positive ion mode (POS): when the substances are ionized in a cation source, the adduct ions are positive ions, such as H+, Na+, K+. ^2^ Negative ion mode (NEG): when the substances are ionized in an ion source, the adduct ions are negative ions, such as Cl−. ^3^ All peaks: number of extracted mass spectrometry peaks.

**Table 2:** Summary of the numbers for differential metabolites among different groups (*p*-value < 0.05 and VIP > 1).

Mode	Comparison Group	Total Differential Metabolites ^1^	Significantly Differential Metabolites	
			Up-Regulated	Down-Regulated
POS	OC vs. BC	687	45	64
	SM vs. BC	1433	130	166
	SM vs. OC	1548	128	175
NEG	OC vs. BC	658	14	49
	SM vs. BC	1646	49	201
	SM vs. OC	1896	64	211

^1^ Total differential metabolites: ion peaks meeting screening criteria.

**Table 3:** Common significantly differential metabolites (SDMs) in different castes of male *M. rosenbergii*.

ID	Metabolites	Metab ID	ID	Metabolites	Metab ID
pos_973	Proline betaine	metab_930	pos_2297	3,4-methylenedioxymethamphetamine	metab_2191
pos_983	Aniline	metab_940	pos_2405	Hexanoyl-L-carnitine	metab_2298
pos_1080	Arsenobetaine	metab_1029	pos_3359	17α-methyltestosterone	metab_3191
pos_1344	Trigonelline	metab_1263	pos_6956	Acrylamide	metab_6582
pos_1358	5,6-methylenedioxy-2-aminoindane	metab_1277	pos_7337	Ethyl 2-{2-[4-(trifluoromethyl)phenyl]hydrazono}propanoate	metab_6935
pos_1414	Glutaconylcarnitine	metab_1332	pos_7489	Creatine	metab_7072
pos_1468	N-nitrosomethylethylamine	metab_1384	neg_3545	20-hydroxy leukotriene B4	metab_11225

**Table 4:** The significantly different metabolites of different male morphotypes (BC, OC, and SM) were classified according to different behaviors.

Behaviors	Ref.	Comparison Group	Metabolites	KEGG Compound Second Category
Aggressive behavior	[35,36]	OC vs. BC	Glutamic acid	Amino acids; Neurotransmitters
		SM vs. OC	Serotonin	Amines; Neurotransmitters
		OC vs. BC	Norepinephrine	Other hormones; Neurotransmitters
Rapid growth	[37,38,39]	SM vs. OC	Glutaconylcarnitine	
		OC vs. BC	Creatine	
		OC vs. BC	3-hydroxyocta-3,6-dienoylcarnitine	
		OC vs. BC	Glu-Glu	
		OC vs. BC	Ile-Val	
		SM vs. BC	L-tyrosine	Amino acids
		SM vs. BC	Nicotinamide adenine dinucleotide	Cofactors
		SM vs. OC	D-sorbitol	Monosaccharides
		OC vs. BC	Glutamic acid	Amino acids; Neurotransmitters
Reproductive development (Sexual behavior)	[40,41]	SM vs. BC	Testosterone	19-Carbon atoms; steroid hormones
		SM vs. BC	Prostaglandin E3	Eicosanoids
Immune response	[42,43,44]	SM vs. BC	Arachidonic acid	Fatty acids
		SM vs. OC	Leukotriene B4	Eicosanoids
		SM vs. OC	5-hydroxyindoleacetylglycine	
		OC vs. BC	Indole-3-acetylglycine	

**Table 5:** Candidate significant differential metabolites (SDMs) that may affect the caste differentiation of male *M. rosenbergii*.

Comparison Group	Metabolites	Mode	*p*_Value	VIP_PLS-DA	Regulate
OC vs. BC	Creatine	POS	0.0202	1.6171	down
OC vs. BC	Glutamic acid	NEG	0.0343	1.1398	down
SM vs. BC	Prostaglandin E3	POS	9.62 × 10^−6^	2.7722	up
SM vs. BC	Arachidonic acid	NEG	0.0005	2.1582	up
SM vs. BC	Testosterone	POS	0.0080	1.8249	up
SM vs. OC	Glutaconylcarnitine	POS	0.0003	2.1287	down
SM vs. OC	Serotonin	POS	0.0314	1.3003	down
SM vs. OC	Glu-Pro	NEG	0.0109	1.1259	down

## Data Availability

Data are available via MetaboLights with identifier MTBLS12610.

## Supplementary Materials

The following supporting information can be downloaded at: https://www.mdpi.com/article/10.3390/ani15131917/s1, Figure S1. Quality control of samples. (A) Positive ion mode. (B) Negative ion mode. The X-axis represents the relative standard deviation (RSD) percentage, the Y-axis represents the cumulative proportion of ion peaks. (C) A Partial Least Squares Discriminant Analysis (PLS-DA). Table S1. Statistical assessment of survival rate, and population structure in *M. rosenbergii* under experimental aquaculture. Table S2 Polynomial regression of weight-length relationships among male morphotypes in *M. rosenbergii.* Table S3 The hierarchical cluster analysis in OC vs. BC. Table S4 The hierarchical cluster analysis in SM vs. BC. Table S5 The hierarchical cluster analysis in SM vs. OC. Table S6 KEGG enrichment network analysis results across three comparative groups.
