# Supplementary material for: Metabolomic Profiling Reveals Social Hierarchy-Specific Metabolite Differences in Male Macrobrachium rosenbergii
Source: Animals (Basel). 2025 Jun 29;15(13):1917. doi: 10.3390/ani15131917 (PMC12249189; doi:10.3390/ani15131917)
Supplement: Supplementary file 1 [file animals-15-01917-s001.zip › Figure S1.pdf]

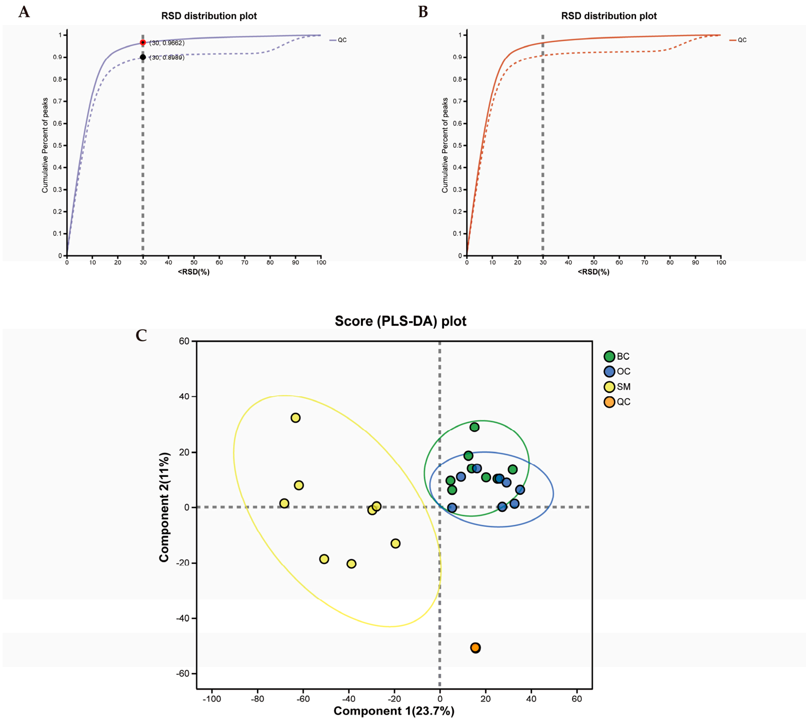

Figure S1. Quality control of samples. (A) Positive ion mode. (B) Negative ion mode. The X-axis represents the relative standard deviation (RSD) percentage, the Y-axis represents the cumulative proportion of ion peaks. (C) A Partial Least Squares Discriminant Analysis (PLS-DA).
