# Supplementary material for: Metabolomic Profiling Reveals Social Hierarchy-Specific Metabolite Differences in Male Macrobrachium rosenbergii
Source: Animals (Basel). 2025 Jun 29;15(13):1917. doi: 10.3390/ani15131917 (PMC12249189; doi:10.3390/ani15131917)
Supplement: Supplementary file 1 [file animals-15-01917-s001.zip › Table S1.pdf]

Table S1. Statistical assessment of survival rate, and population structure in *M. rosenbergii* under experimental aquaculture.

Group	survival rate	Different male morphotypes			Clawless males [1]	Female individuals
		BC	OC	SM		
20-1	50.00%	25 (23.81%)	63 (60.00%)	17 (16.19%)	4	141
20-2	45.80%	18 (20.00%)	56 (62.22%)	16 (17.78%)	0	139
20-3	75.00%	38 (25.33%)	60 (40.00%)	52 (34.67%)	21	204
30-1	43.87%	43 (28.67%)	81 (54.00%)	26 (17.33%)	10	169
30-2	56.67%	40 (23.67%)	82 (48.52%)	47 (27.81%)	26	230
30-3	48.27%	40 (26.32%)	94 (61.84%)	18 (11.84%)	6	204
40-1	46.90%	25 (12.38%)	128 (63.37%)	49 (24.26%)	13	254
40-2	45.10%	53 (26.24%)	104 (51.49%)	45 (22.28%)	9	240
40-3	38.80%	36 (21.05%)	102 (59.65%)	33 (19.30%)	10	207
50-1	41.84%	49 (20.68%)	135 (56.96%)	53 (22.36%)	1	285
50-2	39.36%	47 (23.98%)	110 (56.12%)	39 (19.90%)	3	293
50-3	47.68%	82 (37.10%)	88 (39.82%)	51 (23.08%)	22	353

[1] Clawless male: Chela loss is primarily linked to net harvesting operations, although it may also stem from agonistic behavior under competitive conditions.
