# Supplementary material for: Metabolomic Profiling Reveals Social Hierarchy-Specific Metabolite Differences in Male Macrobrachium rosenbergii
Source: Animals (Basel). 2025 Jun 29;15(13):1917. doi: 10.3390/ani15131917 (PMC12249189; doi:10.3390/ani15131917)
Supplement: Supplementary file 1 [file animals-15-01917-s001.zip › Table S2.pdf]

Table S2 Polynomial regression of weight-length relationships among male morphotypes in *M. rosenbergii.*

Group	term	estimate	std.error	statistic	*p*-value
BC	Intercept	117	0.583	201	1.96E-44
BC	BW_linear	20.9	3.19	6.55	4.98E-07
BC	BW_quadratic	0.137	3.19	0.0428	9.66E-01
OC	Intercept	110	0.363	303	3.09E-49
OC	BW_linear	30.5	1.99	15.4	7.28E-15
OC	BW_quadratic	-2.42	1.99	-1.22	2.34E-01
SM	Intercept	77.5	0.714	109	3.42E-37
SM	BW_linear	34.8	3.91	8.89	1.67E-09
SM	BW_quadratic	-7.82	3.91	-2	5.59E-02
