# Supplementary material for: Metabolomic Profiling Reveals Social Hierarchy-Specific Metabolite Differences in Male Macrobrachium rosenbergii
Source: Animals (Basel). 2025 Jun 29;15(13):1917. doi: 10.3390/ani15131917 (PMC12249189; doi:10.3390/ani15131917)
Supplement: Supplementary file 1 [file animals-15-01917-s001.zip › Table S3-RE.pdf]

Table S3 The hierarchical cluster analysis in OC vs. BC.

Metabolites	Subcluster	Metab ID	Regulate	Mode
Sn-Glycero-3-Phosphocholine	Subcluster1	metab_664	down	pos
Methyl Picolinate	Subcluster1	metab_929	down	pos
Proline Betaine	Subcluster1	metab_930	down	pos
N-[(3S)-2-Oxotetrahydrofuran-3-Yl]Butanamide	Subcluster1	metab_944	down	pos
Choline Glycerophosphate	Subcluster1	metab_1050	down	pos
Glutaconic Acid	Subcluster1	metab_14921	down	neg
3'-Adenylic Acid	Subcluster2	metab_1173	down	pos
Deoxyguanylic Acid	Subcluster2	metab_6535	down	pos
Adenosine 2'-Phosphate	Subcluster2	metab_9177	down	neg
4'-O-Methyldelphinidin 3-O-Beta-D-Glucoside	Subcluster2	metab_14930	down	neg
N-Acetyl-Alpha-D-Galactosamine 1-Phosphate	Subcluster2	metab_14932	down	neg
4-Oxo-L-Proline	Subcluster3	metab_1188	down	pos
N-Acetylaspartic Acid	Subcluster3	metab_9009	down	neg
N-Acetyl-Dl-Glutamic Acid	Subcluster3	metab_14629	down	neg
2,3-Mdma	Subcluster4	metab_1201	down	pos
Leu-Phe	Subcluster4	metab_1711	down	pos
3,4-Edma	Subcluster4	metab_1719	down	pos
8-Amino-7-Oxononanoic Acid	Subcluster4	metab_2039	down	pos
1,3-Benzodioxolylbutanamine	Subcluster4	metab_6373	down	pos
Phenacetine	Subcluster4	metab_6521	down	pos
Deoxycytidine Monophosphate	Subcluster4	metab_8901	down	neg
Deoxyadenosine Monophosphate	Subcluster4	metab_9050	down	neg
4Beta-(2-Aminoethylthio)Epicatechin 3-Gallate	Subcluster4	metab_9070	down	neg
5'-Thymidylic Acid	Subcluster4	metab_14507	down	neg
2'-Deoxyadenosine 3'-Monophosphate	Subcluster4	metab_14730	down	neg

Cmp-2-Aminoethylphosphonate	Subcluster4	metab_14857	down	neg
Glycyl-Lysine	Subcluster5	metab_2008	down	pos
Uridine Monophosphate (Ump)	Subcluster5	metab_9242	down	neg
Uridine Monophosphate	Subcluster5	metab_14874	down	neg
Maltopentose	Subcluster5	metab_15070	down	neg
Leu Glu	Subcluster6	metab_2127	down	pos
Glu Leu	Subcluster6	metab_5806	down	pos
Glu Met	Subcluster6	metab_6216	down	pos
Flavone Base+3O,2Meo,O-Guaiacylglyceryl-Hex	Subcluster6	metab_6896	down	pos
Uridine Diphosphate-N-Acetylgalactosamine	Subcluster6	metab_8782	down	neg
Maltotetraose	Subcluster6	metab_8819	down	neg
2-O-(6-Phospho-Alpha-Mannosyl)-D-Glycerate	Subcluster6	metab_9091	down	neg
Glu-Leu	Subcluster6	metab_10232	down	neg
Thr-Tyr	Subcluster6	metab_13690	down	neg
Gamma-Glu-Leu	Subcluster6	metab_13782	down	neg
Glu-Met	Subcluster6	metab_14341	down	neg
Inosinic Acid	Subcluster6	metab_14594	down	neg
Udp-N-Acetyl-D-Galactosamine	Subcluster6	metab_14803	down	neg
Guanosine 5'-Monophosphate	Subcluster6	metab_14862	down	neg
Inosine 2',3'-Cyclic Phosphate	Subcluster7	metab_9049	up	neg
5-Phenyl-1,3-Oxazinane-2,4-Dione	Subcluster8	metab_10858	up	neg
Emtricitabine	Subcluster8	metab_10859	up	neg
Isoxanthopterin	Subcluster9	metab_14554	up	neg
D-Manno-2-Heptulose	Subcluster10	metab_15122	down	neg
Ala-Gly	Subcluster10	metab_15458	down	neg
