# Supplementary material for: Metabolomic Profiling Reveals Social Hierarchy-Specific Metabolite Differences in Male Macrobrachium rosenbergii
Source: Animals (Basel). 2025 Jun 29;15(13):1917. doi: 10.3390/ani15131917 (PMC12249189; doi:10.3390/ani15131917)
Supplement: Supplementary file 1 [file animals-15-01917-s001.zip › Table S4-RE.pdf]

Table S4 The hierarchical cluster analysis in SM vs. BC.

Metabolites	Subcluster	Metab ID	Regulate	Mode
O-Acetylcarnitine	Subcluster1	metab_1090	up	pos
O-Acetylcarnitinium	Subcluster1	metab_1275	up	pos
Pe(20:5/0:0)	Subcluster1	metab_3230	up	pos
Pc(20:5/0:0)	Subcluster1	metab_3255	up	pos
Lysopc(15:0)	Subcluster1	metab_3311	up	pos
Lpc(17:0)	Subcluster1	metab_3416	up	pos
Pc(18:3/0:0)	Subcluster1	metab_4614	up	pos
Pe(20:4/0:0)	Subcluster1	metab_11335	up	neg
Levonorgestrel Acetate	Subcluster1	metab_12575	up	neg
(+/-)-Propionylcarnitine	Subcluster2	metab_1207	down	pos
4-Coumaric Acid	Subcluster2	metab_1403	down	pos
Octopamine	Subcluster2	metab_1407	down	pos
L-Tyrosine	Subcluster2	metab_1408	down	pos
Propionylcarnitine	Subcluster2	metab_1433	down	pos
2-Hydroxyphenylalanine	Subcluster2	metab_6647	down	pos
L-Phenylalanine	Subcluster2	metab_9609	down	neg
Glu-Leu	Subcluster2	metab_10232	down	neg
(+/-)-Tryptophan	Subcluster2	metab_14007	down	neg
Glu-Met	Subcluster2	metab_14341	down	neg
3-Amino-3-(4-Hydroxyphenyl)Propanoate	Subcluster2	metab_14625	down	neg
Glycyl-Lysine	Subcluster3	metab_2008	down	pos
8-Amino-7-Oxononanoic Acid	Subcluster3	metab_2039	down	pos
Gpcho(17:2/20:2)	Subcluster3	metab_3308	down	pos
Ps(Dime(13,5)/Monome(11,3))	Subcluster3	metab_4182	down	pos
Pe(P-18:0/0:0)	Subcluster4	metab_3483	down	pos
Gpcho(22:6/18:2)	Subcluster4	metab_4166	down	pos
Gpcho(20:5/22:6)	Subcluster4	metab_4282	down	pos

Albafuran A	Subcluster5	metab_3896	up	pos
Fursultiamine	Subcluster5	metab_4137	up	pos
Hypoxanthine	Subcluster5	metab_6279	up	pos
Inosine	Subcluster5	metab_9523	up	neg
Formycin B	Subcluster5	metab_14841	up	neg
Gpcho(18:2/17:0)	Subcluster6	metab_4188	up	pos
Dg(Lte4/I-19:0/0:0)	Subcluster6	metab_4413	up	pos
Pc(Pge2/P-18:1(9Z))	Subcluster6	metab_11411	up	neg
Dihydroxyacetone	Subcluster7	metab_8995	down	neg
Ps(Monome(13,5)/Monome(11,3))	Subcluster7	metab_11360	down	neg
Pc(40:8)	Subcluster7	metab_11606	down	neg
Gpetn(18:2/20:2)	Subcluster7	metab_12556	down	neg
Pe(34:2)	Subcluster7	metab_12579	down	neg
N-Acetyl-Dl-Glutamic Acid	Subcluster7	metab_14629	down	neg
Lactic Acid	Subcluster7	metab_14719	down	neg
Spongouridin	Subcluster8	metab_9325	down	neg
Udp-N-Acetyl-D-Galactosamine	Subcluster8	metab_14803	down	neg
Uridine	Subcluster8	metab_14885	down	neg
Tiglic Aldehyde	Subcluster9	metab_10409	down	neg
Prostaglandin B1	Subcluster9	metab_11340	down	neg
2-Hydroxy-4-Methylvaleric Acid	Subcluster9	metab_13308	down	neg
Uric Acid	Subcluster9	metab_14916	down	neg
6-Geranylnaringenin	Subcluster10	metab_12981	up	neg
