# Supplementary material for: Metabolomic Profiling Reveals Social Hierarchy-Specific Metabolite Differences in Male Macrobrachium rosenbergii
Source: Animals (Basel). 2025 Jun 29;15(13):1917. doi: 10.3390/ani15131917 (PMC12249189; doi:10.3390/ani15131917)
Supplement: Supplementary file 1 [file animals-15-01917-s001.zip › Table S5-RE.pdf]

Table S5 The hierarchical cluster analysis in SM vs. OC.

Metabolites	Subcluster	Metab ID	Regulate	Mode
O-Acetylcarnitine	Subcluster1	metab_1090	up	pos
O-Acetylcarnitinium	Subcluster1	metab_1275	up	pos
Gpcho(16:0/20:5)	Subcluster1	metab_4291	up	pos
Dg(Lte4/I-19:0/0:0)	Subcluster1	metab_4413	up	pos
Pe(20:4/0:0)	Subcluster1	metab_11335	up	neg
Pc(Pge2/P-18:1(9Z))	Subcluster1	metab_11411	up	neg
4-Coumaric Acid	Subcluster2	metab_1403	down	pos
L-Tyrosine	Subcluster2	metab_1408	down	pos
2-Hydroxyphenylalanine	Subcluster2	metab_6647	down	pos
Spongouridin	Subcluster2	metab_9325	down	neg
(+/-)-Tryptophan	Subcluster2	metab_14007	down	neg
3-Amino-3-(4-Hydroxyphenyl)Propanoate	Subcluster2	metab_14625	down	neg
Uridine	Subcluster2	metab_14885	down	neg
Bis-(Acetyl)Diaminopentane	Subcluster3	metab_1923	down	pos
Asp-Ile	Subcluster3	metab_1971	down	pos
Gpcho(22:6/18:2)	Subcluster3	metab_4166	down	pos
Gpetn(18:3/22:5)	Subcluster3	metab_4273	down	pos
Albafuran B	Subcluster4	metab_2928	up	pos
Piperochromanoic Acid	Subcluster4	metab_12973	up	neg
6-Geranylnaringenin	Subcluster4	metab_12981	up	neg
Pc(20:5/0:0)	Subcluster5	metab_3255	up	pos
Lysopc(15:0)	Subcluster5	metab_3311	up	pos
Lpc(17:0)	Subcluster5	metab_3416	up	pos
Albafuran A	Subcluster5	metab_3896	up	pos
Fursultiamine	Subcluster5	metab_4137	up	pos
1,4-Dihydro-2-Methylbenzoicacid	Subcluster5	metab_4296	up	pos
Pc(18:3/0:0)	Subcluster5	metab_4614	up	pos

1-(9Z-Tetradecenoyl)-Glycero-3-Phosphate	Subcluster5	metab_12002	up	neg
Gpcho(20:4/22:6)	Subcluster6	metab_3420	down	pos
Ps(Dime(13,5)/Monome(11,3))	Subcluster6	metab_4182	down	pos
Ps(Monome(13,5)/Monome(11,3))	Subcluster6	metab_11360	down	neg
Pe(36:2)	Subcluster6	metab_11513	down	neg
Pe(Pgf1Alpha/P-18:0)	Subcluster6	metab_11514	down	neg
Gpetn(18:2/20:2)	Subcluster6	metab_12556	down	neg
Pe(34:2)	Subcluster6	metab_12579	down	neg
Hypoxanthine	Subcluster7	metab_6279	up	pos
Phenacetine	Subcluster7	metab_6521	up	pos
Deoxyadenosine Monophosphate	Subcluster7	metab_9050	up	neg
Inosine	Subcluster7	metab_9523	up	neg
5'-Thymidylic Acid	Subcluster7	metab_14507	up	neg
Formycin B	Subcluster7	metab_14841	up	neg
Dihydroxyacetone	Subcluster8	metab_8995	down	neg
Tiglic Aldehyde	Subcluster8	metab_10409	down	neg
Prostaglandin B1	Subcluster8	metab_11340	down	neg
Pc(40:8)	Subcluster8	metab_11606	down	neg
Lactic Acid	Subcluster8	metab_14719	down	neg
Uric Acid	Subcluster8	metab_14916	down	neg
Pe-Nme2(18:1(9Z)/18:1(9Z))	Subcluster9	metab_12486	down	neg
3,13-Dihydroxytetradecanoylcarnitine	Subcluster9	metab_12578	down	neg
Citric Acid	Subcluster10	metab_14908	up	neg
