# Supplementary material for: Metabolomic Profiling Reveals Social Hierarchy-Specific Metabolite Differences in Male Macrobrachium rosenbergii
Source: Animals (Basel). 2025 Jun 29;15(13):1917. doi: 10.3390/ani15131917 (PMC12249189; doi:10.3390/ani15131917)
Supplement: Supplementary file 1 [file animals-15-01917-s001.zip › Table S6-RE.pdf]

Table S6 KEGG enrichment network analysis results across three comparative groups.

Comparison group	Pathway ID	Pathway Desciption	*p*_value	Metabolites
OC vs. BC	map01240	Biosynthesis of cofactors	0.0017	P-Aminobenzoic Acid; 8-Amino-7-Oxononanoic Acid; 5-Amino-6-(5'-Phosphoribitylamino)Uracil; Desthiobiotin; Orotic Acid; D-Ribose 5-Phosphate; Niacinamide; Dihydroneopterin Triphosphate; Inosinic Acid; Uridine Monophosphate; Adenosine Monophosphate; Glutamic Acid
OC vs. BC	map00440	Phosphonate and phosphinate metabolism	0.0020	D-Ribose 5-Phosphate; Bialaphos; Cmp-2-Aminoethylphosphonate
OC vs. BC	map04742	Taste transduction	0.0027	Norepinephrine; Serotonin; Inosinic Acid; Guanosine 5'-Monophosphate; Adenosine Monophosphate; Glutamic Acid
OC vs. BC	map01523	Antifolate resistance	0.0029	5'-Thymidylic Acid; Inosinic Acid; Guanosine 5'-Monophosphate; Adenosine Monophosphate
OC vs. BC	map01232	Nucleotide metabolism	0.0043	Deoxyguanylic Acid; Deoxycytidine Monophosphate; Deoxyadenosine Monophosphate; 5'-Thymidylic Acid; Inosinic Acid; Guanosine 5'-Monophosphate; Uridine Monophosphate; Adenosine Monophosphate
OC vs. BC	map04721	Synaptic vesicle cycle	0.0072	Norepinephrine; Serotonin; Glutamic Acid
OC vs. BC	map04068	FoxO signaling pathway	0.0160	Adenosine Monophosphate; Glutamic Acid
OC vs. BC	map04540	Gap junction	0.0163	Norepinephrine; Serotonin; Glutamic Acid
OC vs. BC	map00790	Folate biosynthesis	0.0163	P-Aminobenzoic Acid; 6-Lactoyltetrahydropterin; Dihydroneopterin Triphosphate
OC vs. BC	map00480	Glutathione metabolism	0.0163	L-Ornithine; 5-L-Glutamyl-L-Alanine; Glutamic Acid
OC vs. BC	map00230	Purine metabolism	0.0175	3'-Adenylic Acid; Deoxyguanylic Acid; Deoxyadenosine Monophosphate; D-Ribose 5-Phosphate; Inosinic Acid; Guanosine 5'-Monophosphate; Adenosine Monophosphate

OC vs. BC	map00240	Pyrimidine metabolism	0.0307	3-Ureidopropionic Acid; Deoxycytidine Monophosphate; Orotic Acid; 5'-Thymidylic Acid; Uridine Monophosphate
OC vs. BC	map05022	Pathways of neurodegeneration - multiple diseases	0.0441	Adenosine Monophosphate; Glutamic Acid
OC vs. BC	map00030	Pentose phosphate pathway	0.0441	D-Ribose 5-Phosphate; Deoxyribose 5-Phosphate
OC vs. BC	map04024	cAMP signaling pathway	0.0690	Norepinephrine; Serotonin; Adenosine Monophosphate
OC vs. BC	map00780	Biotin metabolism	0.0811	8-Amino-7-Oxononanoic Acid; Desthiobiotin
OC vs. BC	map04022	cGMP-PKG signaling pathway	0.0811	Guanosine 5'-Monophosphate; Adenosine Monophosphate
OC vs. BC	map04740	Olfactory transduction	0.0811	Guanosine 5'-Monophosphate; Adenosine Monophosphate
OC vs. BC	map04924	Renin secretion	0.1242	Norepinephrine; Adenosine Monophosphate
OC vs. BC	map05207	Chemical carcinogenesis - receptor activation	0.1242	Norepinephrine; Serotonin
SM vs. BC	map00730	Thiamine metabolism	0.0845	Nicotinamide Adenine Dinucleotide; L-Tyrosine
SM vs. BC	map04020	Calcium signaling pathway	0.0845	Cyclic Adp-Ribose; Acrasin
SM vs. BC	map04725	Cholinergic synapse	0.0845	Acetylcholine; Acrasin
SM vs. BC	map04911	Insulin secretion	0.0845	Acetylcholine; Acrasin
SM vs. BC	map04916	Melanogenesis	0.0845	L-Tyrosine; Acrasin
SM vs. BC	map04971	Gastric acid secretion	0.0845	Acetylcholine; Acrasin
SM vs. BC	map01522	Endocrine resistance	0.0845	Testosterone; Acrasin

SM vs. BC	map04912	GnRH signaling pathway	0.0845	Acrasin; Arachidonic Acid
SM vs. BC	map05146	Amoebiasis	0.0845	Acrasin; Arachidonic Acid
SM vs. BC	map03320	PPAR signaling pathway	0.0845	Leukotriene B4; 8-Hete
SM vs. BC	map04972	Pancreatic secretion	0.0243	Acetylcholine; Cyclic Adp-Ribose; Acrasin
SM vs. BC	map00040	Pentose and glucuronate interconversions	0.0764	D-Lyxose; 2-Oxoglutaric Acid; Tyramine Glucuronide
SM vs. BC	map04921	Oxytocin signaling pathway	0.0764	Cyclic Adp-Ribose; Acrasin; Arachidonic Acid
SM vs. BC	map04913	Ovarian steroidogenesis	0.0069	Testosterone; 11,12-Epoxyeicosatrienoic Acid; Acrasin; Arachidonic Acid
SM vs. BC	map00740	Riboflavin metabolism	0.0624	Lumichrome; Riboflavin; Flavin Mononucleotide; Riboflavin Reduced
SM vs. BC	map00982	Drug metabolism - cytochrome P450	0.1133	Cyclophosphamide; Morphine-6-Glucuronide; Morphine-3-Glucuronide; Codeine-6-Glucuronide
SM vs. BC	map04723	Retrograde endocannabinoid signaling	0.0624	Gpcho(18:4/14:1); Gpcho(22:6/18:2); Gpetn(18:3/22:4); Acrasin; Arachidonic Acid
SM vs. BC	map04726	Serotonergic synapse	0.0862	11,12-Epoxyeicosatrienoic Acid; 5-Hydroxyindoleacetic Acid; Acrasin; Leukotriene B4; Arachidonic Acid
SM vs. BC	map00360	Phenylalanine metabolism	0.1013	2-Hydroxycinnamic Acid; L-Tyrosine; 2-Phenylethylamine; L-Phenylalanine; M-Coumaric Acid; Phenylpyruvic Acid
SM vs. BC	map00590	Arachidonic acid metabolism	0.0309	15-Deoxy-Delta-12,14-Prostaglandin J2; Gpcho(18:4/14:1); Gpcho(22:6/18:2); 11,12-Epoxyeicosatrienoic Acid; 20-Hydroxy Leukotriene B4; Leukotriene B4; Arachidonic Acid; 8-Hete
SM vs. OC	map04917	Prolactin signaling pathway	0.1032	L-Tyrosine; L-Dopa
SM vs. OC	map03320	PPAR signaling pathway	0.1032	Leukotriene B4; 8-Hete

SM vs. OC	map00040	Pentose and glucuronate interconversions	0.1003	D-Lyxose; 2-Oxoglutaric Acid; Tyramine Glucuronide
SM vs. OC	map00785	Lipoic acid metabolism	0.1003	2-Oxoglutaric Acid; 2-Oxoadipic Acid; 3-Methyl-2-Oxovaleric Acid
SM vs. OC	map05012	Parkinson disease	0.1003	L-Tyrosine; L-Dopa; Adenosine Monophosphate
SM vs. OC	map04913	Ovarian steroidogenesis	0.1003	Testosterone; 11,12-Epoxyeicosatrienoic Acid; Arachidonic Acid
SM vs. OC	map05033	Nicotine addiction	0.1032	Glutamate; Acetylcholine; Glutamic Acid
SM vs. OC	map04068	FoxO signaling pathway	0.1032	Glutamate; Adenosine Monophosphate; Glutamic Acid
SM vs. OC	map05022	Pathways of neurodegeneration - multiple diseases	0.0329	Glutamate; Acetylcholine; Adenosine Monophosphate; Glutamic Acid
SM vs. OC	map04721	Synaptic vesicle cycle	0.1003	Serotonin; Glutamate; Acetylcholine; Glutamic Acid
SM vs. OC	map05030	Cocaine addiction	0.0389	L-Tyrosine; Glutamate; L-Dopa; 3,4-Dihydroxyphenylacetic Acid; Glutamic Acid
SM vs. OC	map05031	Amphetamine addiction	0.0389	L-Tyrosine; Glutamate; L-Dopa; 3,4-Dihydroxyphenylacetic Acid; Glutamic Acid
SM vs. OC	map05034	Alcoholism	0.0389	L-Tyrosine; Glutamate; L-Dopa; 3,4-Dihydroxyphenylacetic Acid; Glutamic Acid
SM vs. OC	map00400	Phenylalanine, tyrosine and tryptophan biosynthesis	0.0747	L-Tyrosine; Anthranilic Acid; (1S,2R)-1-C-(Indol-3-Yl)Glycerol 3-Phosphate; Pretyrosine; Phenylpyruvic Acid
SM vs. OC	map00310	Lysine degradation	0.0695	N6,N6,N6-Trimethyl-L-Lysine; Pipecolic Acid; N-Epsilon-Acetyl-L-Lysine; L-Lysine; 2-Oxoglutaric Acid; 2-Oxoadipic Acid; Glutaric Acid; 5-Aminovaleric Acid
SM vs. OC	map00590	Arachidonic acid metabolism	0.0529	15-Deoxy-Delta-12,14-Prostaglandin J2; Gpcho(18:4/14:1); Gpcho(22:6/18:2); Gpcho(16:0/20:5); 11,12-Epoxyeicosatrienoic Acid; 20-Hydroxy Leukotriene B4; Leukotriene B4; Arachidonic Acid; 8-Hete

SM vs. OC	map04723	Retrograde endocannabinoid signaling	0.0877	Gpcho(18:4/14:1); Gpcho(22:6/18:2); Gpetn(18:3/22:4); Gpcho(16:0/20:5); Glutamate; Pe(36:1); Pe(36:2); Arachidonic Acid; Glutamic Acid
SM vs. OC	map00380	Tryptophan metabolism	0.0393	Kynurenic Acid; N-Acetyl-5-Hydroxytryptamine; 3-Methylindole; Serotonin; 5-Hydroxyindoleacetylglycine; Anthranilic Acid; 3-Hydroxyanthranilic Acid; 5-Hydroxyindoleacetic Acid; 2-Oxoadipic Acid; 2-Formamidobenzoic Acid; Tryptamine
SM vs. OC	map00564	Glycerophospholipid metabolism	0.0747	Pc(22:5/0:0); Lysopc(15:0); Lpc(17:0); Gpcho(18:4/14:1); Gpcho(22:6/18:2); Gpetn(18:3/22:4); Gpcho(16:0/20:5); Acetylcholine; Pe(36:1); Pe(36:2); Pe-Nme2(18:1(9Z)/18:1(9Z))
SM vs. OC	map01232	Nucleotide metabolism	0.0113	Guanine; Deoxyadenosine; Thymine; Hypoxanthine; Deoxyguanylic Acid; Deoxycytidine Monophosphate; Deoxyadenosine Monophosphate; Inosine; Thymidine; Deoxyinosine; 5'-Thymidylic Acid; Uridine; Adenosine Monophosphate
